# Shape Memory Respirator Mask for Airborne Viruses

**DOI:** 10.3390/polym15081859

**Published:** 2023-04-13

**Authors:** Kosisochi Ibebunjo, Susanna Tella, Samantha Kiljunen, Eveliina Repo

**Affiliations:** 1 Department of Separation Science, School of Engineering Science, LUT University, FI-53850 Lappeenranta, Finland; 2 Faculty of Health Care and Social Services, LAB University of Applied Sciences, FI-53850 Lappeenranta, Finland

**Keywords:** respirator, facemask, shape memory polymer, SMP, PLA, PCL, polymer extrusion, 3D printing

## Abstract

The emergence of COVID-19 has spurred demand for facemasks and prompted many studies aiming to develop masks that provide maximum protection. Filtration capacity and fit define the level of protection a mask can provide, and the fit is in large part determined by face shape and size. Due to differences in face dimensions and shapes, a mask of one size will not be likely to fit all faces. In this work, we examined shape memory polymers (SMPs) for producing facemasks that are able to alter their shape and size to fit every face. Polymer blends with and without additives or compatibilizers were melt-extruded, and their morphology, melting and crystallization behavior, mechanical properties, and shape memory (SM) behavior were characterized. All the blends had phase-separated morphology. The mechanical properties of the SMPs were modified by altering the content of polymers and compatibilizers or additives in the blends. The reversible and fixing phases are determined by the melting transitions. SM behavior is caused by physical interaction at the interface between the two phases in the blend and the crystallization of the reversible phase. The optimal SM blend and printing material for the mask was determined to be a polylactic acid (PLA)/polycaprolactone (PCL) blend with 30% PCL. A 3D-printed respirator mask was manufactured and fitted to several faces after being thermally activated at 65°C. The mask had excellent SM and could be molded and remolded to fit a variety of facial shapes and sizes. The mask also exhibited self-healing and healed from surface scratches.

## 1. Introduction

The demand for facemasks has increased dramatically following the emergence of a novel coronavirus in 2019 [1]. The mode of transmission of this virus can be by contact transmission, droplet transmission, and airborne [1,2]. To prevent the virus from spreading, social distancing, good personal hygiene, and self-quarantine were recommended and implemented. Lockdown and travel restrictions were also implemented, resulting in a slowdown in the economy [1,3]. Facemasks were advocated by the Centers for Disease Control and Prevention (CDC) and the World Health Organization (WHO) to limit the spread of the virus and save lives [4,5].

Facemasks have long been recognized as an important form of personal protective equipment (PPE) and are used to filter contaminated air before it is inhaled, as well as to prevent contamination of the environment after someone sneezes or coughs [3]. The type of material used in mask construction and its ability to fit properly and prevent leakage from the sides determines the quality and effectiveness of the mask [3,4]. Medical masks and respirators are considered the best means of protecting against viruses in public or healthcare settings [6]. Layers of nonwoven material made from polymers such as polystyrene, polyethylene, and polypropylene are generally utilized to make medical and respirator masks using electrospinning, melt-blowing, and spin-bonding methods [1,7]. Another technology that has been employed to produce facemasks, particularly during the COVID-19 pandemic, is 3D printing. This technology can be used to print a variety of mask designs as well as personalized masks based on the wearer’s facial dimensions [8].

Medical masks are designed to be loose-fitting and may not provide complete protection against infections. Respirators, on the other hand, are designed to be tight-fitting and provide a better level of infection protection [9]. Although respirator masks are tight-fitting, a wearer’s ability to find a mask that fits well is determined by his or her facial dimensions. Face sizes and shapes vary, and thus one-size-fits-all respirator masks do not fit everyone [10]. A mask can, of course, be customized to fit a person’s face dimensions and size, but it can then only be worn by that person and will not precisely fit another face. Customized respirator masks are limited in their usefulness because they cannot be altered to fit another person for reuse, which generates waste and increases demand for new masks.

Shape memory polymers (SMPs) have been proposed by Bailar et al. [10] as an alternative material for producing facemasks, but to the authors’ best knowledge, no research into this concept has yet been reported. SMPs are stimuli-responsive polymers that can shift to a temporary shape from their permanent shape when exposed to stimuli such as light, heat, and magnetic fields and then return to their original shape [11,12,13,14]. SMPs are made up of a soft segment and a hard segment, where the soft segment functions as a temporary shape and is responsible for changing shape, whilst the hard segment is responsible for defining the permanent shape [11]. The temporary shape is obtained after deforming the permanent shape by a programming procedure of heating, deforming, and cooling, or by cold drawing. Following programming, the polymer displays its temporary shape while remembering its permanent shape. The temporary shape can revert to the permanent shape when exposed to stimuli such as heating to temperatures higher than its transition temperature (T_trans_) [14,15].

Shape memory polymers respond to various stimuli; those responsive to temperature are termed thermo-responsive SMPs. Their shape memory effect (SME) is a result of combined thermo-mechanical programming and polymer morphology [12]. The thermo-responsive SMP is shaped when it is heated above the melting temperature (T_m_) or glass transition temperature (T_g_) of the hard segment, and on subsequent cooling below the T_m_ or T_g_ of the hard segment, the permanent shape is created and memorized. The temporary shape is created when the permanent shape is deformed below the T_m_ or T_g_ of the hard segment but above the T_m_ or T_g_ of the soft segment. The deformed polymer is then cooled below the T_m_ or T_g_ of the soft segment to fix the temporary shape. To return to its permanent shape, the deformed polymer is reheated above the T_m_ or T_g_ of the soft segment [16]. Several thermo-responsive SMPs have been reported [13,17,18,19,20]. SMPs can also repair mechanical damage such as scratches and cracks. This is known as self-healing behavior, and it occurs because of their shape recovery, which causes their cracked or scratched surfaces to move closer together when activated by an external stimulus such as heat. This is called reversible plasticity SME and can increase the polymer’s lifespan and performance [21,22,23]. Overall, SMPs have unique properties that make them potentially valuable materials for a variety of challenging applications such as self-healing materials, drug carriers, smart medical devices, and fabrics [12].

Thermo-responsive SMPs are manufactured by physically blending two polymers, where one polymer has a higher T_m_ or T_g_ than the other. The polymers can be either immiscible or miscible, and blending can also involve a tertiary component such as a crosslinker or a compatibilizer to enhance their properties [12]. Shape memory has been reported in a miscible blend of thermoplastic polyurethane (TPU) and polyvinylchloride (PVC), and also in an immiscible poly(p-dioxanone) (PPDO)/polycaprolactone (PCL) blend [24] and polylactic acid (PLA)/polycaprolactone (PCL) blends [17,19].

This study explores the use of shape memory polymers (SMPs) for producing respirator masks. Thermo-responsive SMPs consisting of two polymers and those consisting of two polymers with additives or compatibilizers were melt-extruded. The prepared SMPs were characterized in terms of mechanical properties, morphology, melting and crystallization behavior, shape memory behavior, and self-healing. Subsequently, the extruded filaments were used as 3D-printing materials for respirator masks that can alter their shape and size to fit every face when heated. The feasibility of the approach was tested by affixing the masks to different faces.

## 2. Materials and Methods

### 2.1. Materials

The materials used in this work and their properties are given in Table 1 below.

### 2.2. Blend Preparation

Before melt blending, the polymer pellets were dried for 12 h in an oven set at 40 °C and then premixed in a sealed bag to ensure a uniform mixture. The weight ratios of the premixed polymers were as follows: PP/PCL (10–30 wt.%); PP/PCL (10–30 wt.%)/PP-g-ma (1.25–10 phr); PLA/PCL (10–60 wt.%); PLA/PCL (20–40 wt.%)/P-123 (2.5–10 phr); LDPE/PCL (10–30 wt.%); LDPE/PCL (10–30 wt.%)/PE-g-ma (10 phr). The premixed polymers were made into filaments using a Filabot EX2 extruder, operating at temperatures of 20–30 °C above the melting temperatures provided by the supplier. Neat PLA, PCL, LDPE, and PP, denoted as PLA100, PCL100, LDPE100, and PP100, respectively, were also extruded for comparison.

### 2.3. Characterization Techniques

#### 2.3.1. Tensile Testing

The mechanical properties of the pristine polymers and their blends were assessed using a universal testing machine (Zwick Roell Z020, Zwick Roell group, Ulm, Germany) operating with a crosshead speed of 30 mm/min. Each filament (blend) was sliced into 165 mm long test samples and conditioned for 72 h in a conditioning chamber at a temperature and relative humidity of 23 °C and 65%, respectively. The tests were performed at room temperature using a minimum of five samples of each blend. The Young’s modulus, elongation at break, and tensile strength were obtained from the mean values of the tested samples.

#### 2.3.2. Differential Scanning Calorimetry (DSC)

A NETZSCH DSC 204FI differential scanning calorimeter was used to investigate the melting and crystallization behavior of the blends. The melting temperature (T_m_), glass transition temperature (T_g_), crystallization and melting enthalpy, and crystallization temperature of the blends were investigated. The experiment consisted of three stages performed at a heating rate of 10 K/min under nitrogen atmosphere. Samples were heated from 20 °C to 200 °C, and then they were cooled to –50°C and heated once more from –50 °C to 200 °C. The degree of crystallization (*X_c_*) of LDPE (*X_c_*_, LDPE_), PCL (*X_c_*_, PCL_), and PP (*X_c_*_, PP_) was calculated using Equation (1), and that of PLA (*X_c_*_, PLA_) was calculated using Equation (2). Melting enthalpy at 100% crystallization was assumed as 93.6 J/g for PLA [17] and 293 J/g, 136 J/g, and 205 J/g for PCL, LDPE, and PP, respectively [17,25].

(1) $$X_{c}\left(\%\right)=100\%\;\times \left[\frac{\Delta H_{m}}{\Delta H_{m}^{o}\times W}\right]$$

(2) $$X_{c}\left(\%\right)=100\%\;\times \left[\frac{\Delta H_{m}-\Delta H_{cc}}{\Delta H_{m}^{o}\times W}\right]$$

where $\Delta H_{m}$ is the melting enthalpy, $\Delta H_{m}^{o}$ is the crystalline melting enthalpy at 100%, $\Delta H_{cc}$ is the cold crystallization enthalpy, and W is the weight content of PCL, LDPE, PCL, and PP in the blends.

#### 2.3.3. Scanning Electron Microscopy (SEM)

A Hitachi SU3500 scanning electron microscope with a 5 kV accelerating voltage was used to study the morphologies of the blends. The test samples fractured following immersion in liquid nitrogen for 3–5 min (cryogenic fracture) were put on a specimen stub, spray-coated with gold/palladium, and then subjected to SEM for imaging.

#### 2.3.4. Shape Memory Test

Thermomechanical tests were conducted manually to study the shape memory behavior of the blends. Each filament (blend) was sliced into test specimens of 18 cm in length. The specimens were first heated in an oven at a deformation temperature (T_d_) of 65 °C for 10 min. The initial strain, $\varepsilon_{p}\left(N-1\right)$, was recorded. Afterward, the heated specimens were held at a temporary strain ($\varepsilon_{m}$) of 15, 25, and 50% for 3 min, and the temporary shape was fixed by allowing the sample to cool to room temperature. The strain applied was withdrawn, and the new strain, $\varepsilon_{u}$, was recorded after five minutes. The permanent shape was recovered by heating the specimen at T_d_ for 10–20 min. The initial shape was restored, and the final strain, $\varepsilon_{p}\left(N\right)$, was recorded. The shape recovery (*R_r_*) was calculated using Equation (3), and the shape fixity (*R_f_*) was calculated using Equation (4) [14].

(3) $$R_{r}=\frac{\varepsilon_{m}-\varepsilon_{p}\left(N\right)}{\epsilon_{m}-\varepsilon_{p}\left(N-1\right)}\times 100\%$$

(4) $$R_{f}=\frac{\varepsilon_{u}\left(N\right)}{\epsilon_{m}}\times 100\%$$

### 2.4. Three-Dimensional Printing

The 3D model of the respirator mask was downloaded from MakerBot Thingiverse [26]. The mask was created using Prenta Duo FDM printer. The 3D model was first put into slicing software (Slic3r), where printing settings were modified to develop a G-code for the model. The extruded filaments were then fed into the FDM printer, which manufactured the 3D model by scanning around the printing bed with the heated nozzle corresponding to the positions given by the G-code. The mask had four parts: the cap, the filter holder, the filter connector, and the mask body. The cap, filter holder, and connector were 3D-printed with pristine filament, whereas the body was 3D-printed with an extruded filament of blends that showed shape memory.

## 3. Results and Discussion

### 3.1. Polymer Extrusion

PLA/PCL, PP/PCL, LDPE/PCL, PP/PCL/PP-g-ma, and LDPE/PCL/PE-g-ma polymer blends were extruded into homogeneous filaments that were then processed. PLA/PCL/P-123 polymer blends were extruded into homogeneous filaments for 2.5–5 phr additive concentration; however, the produced filaments became less homogeneous as the additive level increased.

### 3.2. Mechanical Properties

**Figure 1:**
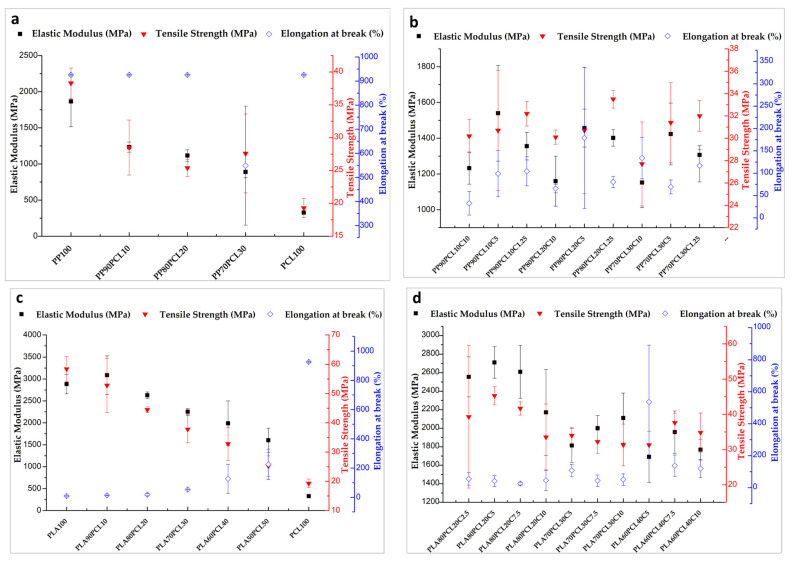

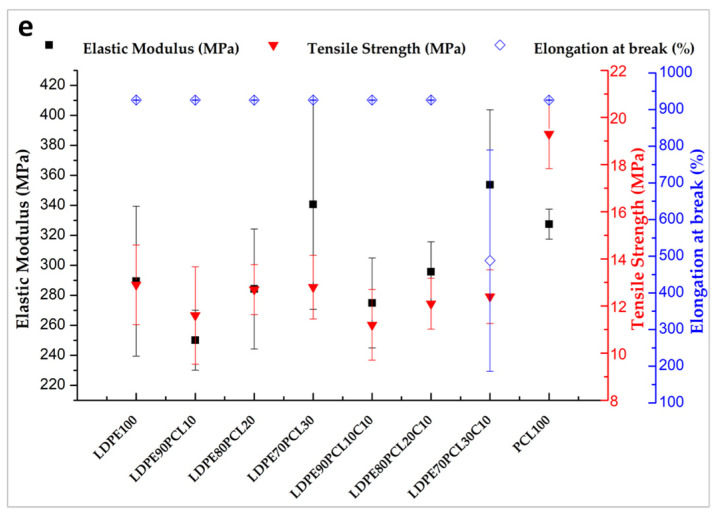
Stress–strain plots of (**a**) PP/PCL, (**b**) PP/PCL/PP-g-ma, (**c**) PLA/PCL, (**d**) PLA/PCL/ P-123, and (**e**) LDPE/PCL and LDPE/PCL/PE-g-ma blends.

Figure 1a,b present the stress–strain plots of the PP/PCL and PP/PCL/PP-g-ma blends, and Tables S1 and S2 (Supporting Information) summarize the mechanical properties of the blends as determined from the plots. PP100 has excellent elastic modulus and tensile strength. PCL100, however, has poor tensile strength and elastic modulus. The tensile strength of PP100 was 38.3 MPa, and that of PCL100 was 19.3 MPa, as shown in Figure 1a. Both polymers showed excellent ductility, with elongation at break of over 926%. The inclusion of PCL in PP altered its tensile behavior and decreased its elastic modulus and tensile strength. The elongation at break remained constant for 10–20 wt.% PCL but reduced for 30 wt.% PCL. The tensile strength and elastic modulus decreased with the inclusion of PCL because PCL has a lower elastic modulus and tensile strength than PP. PP-g-ma, which has been used as an effective polyolefin blend compatibilizer [27], was blended with PP/PCL to increase the miscibility of the blend as well as the material properties. The PP-g-ma content in the blend was varied between 1.25 and 10 phr. Figure 1b shows that PP-g-ma enhanced the tensile strength and elastic modulus of the blends when compared to the blends without PP-g-ma enhancement, although it significantly reduced their ductility. In addition, the blends with 1.25 phr of the compatibilizer had better tensile strength and ductility than the blends with 5 and 10 phr.

The stress–strain plots of PLA/PCL and PLA/PCL/P-123 blends are presented in Figure 1c,d and a summary of their mechanical properties is given in Tables S3 and S4 (Supporting Information). As shown in Figure 1c, PLA100 exhibited excellent elastic modulus and tensile strength, whereas PCL100 exhibited low elastic modulus and tensile strength. The tensile strength of PLA100 was 58.4 MPa, and that of PCL100 was 19.3 MPa, at break. PCL showed excellent ductility with elongation at break of over 926%, while that of PLA was poor at 8.62%. The addition of 10–50 wt.% PCL to PLA altered its tensile properties, and the tensile strength and elastic modulus decreased as the PCL content increased, displaying the plasticizing effect PCL has on PLA as reported by Navarro-Baena et al. [19]. The tensile strength and elastic modulus decreased as the PCL content increased because PCL has a lower elastic modulus and tensile strength than PLA. The inclusion of PCL in PLA increased the ductility with increasing PCL content, showing that PCL helps PLA become less brittle. Ferri et al. [28] reported a similar result, which they attributed to the plasticization effect of PCL on the PLA/PCL matrix. To further improve the ductility of the PLA/PCL blend, P-123 which ranged from 2.5 phr to 10 phr was introduced to the PLA/PCL (20–40 wt.%) blend as an additive. The effect of P-123 as a plasticizer on PLA/PCL blends was previously reported earlier by Wachirahuttapong et al. [29]. As can be seen in Figure 1d, P-123 enhanced the ductility of the blends but reduced their tensile strength and elastic modulus. The addition of P-123 resulted in different behavior with 80PLA/20PCL5, which had a somewhat higher tensile strength and elastic modulus than 80PLA/20PCL. Elongation at break was irregular in the 70PLA/30PCL and 80PLA/20PCL blend with P-123 but followed a trend in 60PLA/40PCL blend with P-123. When blends without additive and blends with additive were compared, the greatest elongation at break was obtained with the additive content of 2.5 phr in the 80PLA/20PCL blend and 5 phr in the 60PLA/40PCL and 70PLA/30PCL blends. This result shows that lower additive concentration favored ductility more than higher content. These results are contrary to the findings reported by Wachirahuttapong et al. [29], who found that the ductility of PLA/PCL blends rose with increasing P-123 content. The conflicting findings can be because of size and temperature differences resulting in an inappropriate blending of the polymers and additives in the extruder, causing excess P-123 to settle and extrude separately from the bulk polymer.

Figure 1e shows the stress–strain plots of the LDPE/PCL and LDPE/PCL/PE-g-ma blends. The mechanical properties are summarized in Table S5 (Supporting Information). Both LDPE100 and PCL100 have poor elastic modulus and tensile strength but excellent ductility, with elongation at break of over 926%. The tensile strength of LDPE100 was 12.9 MPa, and that of PCL100 was 19.3 MPa. The inclusion of PCL in LDPE increased its elastic modulus. The tensile strength did not differ greatly, and the ductility was maintained. The LDPE70/PCL30 blend had a higher elastic modulus than the LDPE100, LDPE90/PCL10, and LDPE80/PCL20 blends because of the higher PCL content. PE-g-ma, which has been used as an effective polyolefin blend compatibilizer [27], was blended with LDPE/PCL to increase the miscibility of the blend as well as the material properties. The PE-g-ma content in the blend was 10 phr for all blends. PE-g-ma marginally increased the elastic modulus and decreased the tensile strength slightly when compared to blends without PE-g-ma. The ductility values of LDPE90/PCL10C and LDPE80/PCL20C were both above 926%, while that of LDPE70/PCL30C was drastically reduced to 488%. It is possible that this is due to a compatibilization issue.

### 3.3. Filament Testing

LDPE/PCL blends were difficult to 3D print, so they were screened out of the manufacturing process. The printing problems of LDPE/PCL blends were due to the low tensile strength, which caused the filament to bend beneath the feeding wheels even at extremely low printing speeds. Results obtained from DSC and SEM are given in Figures S1 and S2, and Table S6 (Supporting Information), respectively. The PP/PCL blends were unable to adhere to the printing bed during printing, resulting in warping. Additional adhesion was applied by using double-sided PP tape on the printing bed to accomplish successful printing. PLA/PCL was 3D-printed without challenges.

### 3.4. Melting and Crystallisation Behaviors

**Figure 2:**
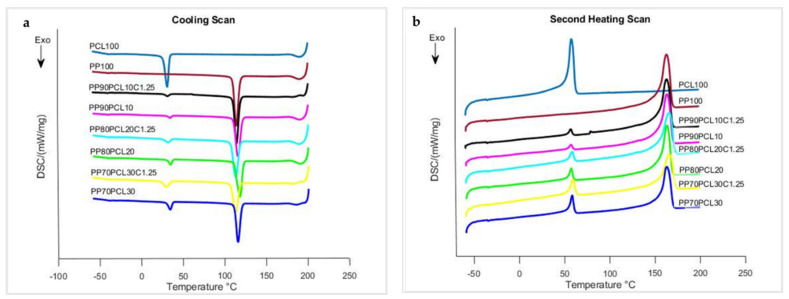
DSC plots of PP, PCL, PP/PCL and PP/PCL compatibilized blends: (**a**) cooling scans and (**b**) second heating scans.

To characterize the melting and crystallization behavior, suitable blends were selected based on their mechanical properties. Pure polymers (PP100, PLA100, PCL100) were also characterized. Results obtained from DSC for PP100, PCL100, and the blends selected are shown in Figure 2a,b as cooling and heating scans, respectively, while their thermal parameters obtained from DSC plots are listed in Table 2. PCL100 and PP100 displayed melt crystallization (T_mc_) peaks at 32.3 °C and 115.2 °C (Figure 2a) and displayed melting temperatures (T_m_) at 58.6 °C and 163.8 °C (Figure 2b). T_mc, PCL_ decreased a little from 35.4 °C for 10 wt.% PCL content to 34.3 °C for 30 wt.% PCL content. These values clearly differed from the T_mc, PCL_ of PCL100. T_m, PCL_ and T_m, PP_ did not change much with varying PCL content and stayed at about 59 °C and 163.8 °C, respectively, although T_mc, PP_ for PP80PCL20 moved marginally.

The lack of or only minor changes in PP and PCL thermal transitions in their blends indicates weak interaction between the two polymers [28]. As given in Table 2, the melt crystallization enthalpy (*H_mc_*) of PCL100 and PP100 was 57.3 Jg^−1^ and 82.6 Jg^−1^, respectively, while their melting enthalpy (*H_m_*) was 65.8 Jg^−1^ and 78.3 Jg^−1^. The *H_mc_*_, PCL_ and *H_m_*_, PCL_ in PP/PCL blends increased as the PCL content increased, but the *H_mc_*_, PP_ and *H_m_*_, PP_ in the blends decreased as the PCL content increased. The degree of crystallization (*X_c_*) of PCL100, PP100, and the blends was calculated using Equation (1). PCL100 and PP100 exhibited excellent crystallization behavior with *X_c_* of 48.38% and 38.21%, respectively. The *X_c_*_, PCL_ in blends decreased when contrasted with PCL100. The *X_c_*_, PCL_ increased from 15.4% for PP90PCL10 to 27.6% for PP70PCL30, showing that low PCL concentration in blends inhibited crystallization of the PCL segment. The *X_c_*_, PP_ in the blends increased when compared to PP100, showing that PCL enhanced the crystallization behavior of the PP segment even more. PP80PCL20 had the highest *X_c_*, whereas the PP90PCL10 and PP70PCL30 blends had somewhat lower *X_c_*, indicating that adding 20 wt.% PCL improved the crystallization of the PP segment slightly. Figure 2 and Table 2 also show the results of the compatibilized blend. T_mc, PCL_ (Figure 2a) and T_m, PCL_ (Figure 2b) did not change considerably with changing PCL content and inclusion of PP-g-ma and remained at approximately 32 °C and 59 °C, respectively. T_mc, PP_ reduced a little from 115.2 °C for PP100 to 114.2 °C and 113.2 °C for PP80PCL20C1.25 and PP70PCL3C1.25, respectively, while T_m, PP_ visibly increased from 163.8 °C for PP100 to about 166 °C for both PP80PCL20C1.25 and PP70PCL3C1.25 blends. These tiny variations in the thermal transitions of PCL and PP in PP/PCL/PP-g-ma blends indicate increased interfacial interaction between both polymers and possibly better miscibility. As reported in Table 2, the *H_mc_* and *H_m_* of PCL in PP/PCL/PP-g-ma blends increased as the PCL content increased, but the *H_mc_* and *H_m_* of PP in the blends decreased as the PCL content increased. The addition of a compatibilizer, PP-g-ma, significantly improved the crystallization behavior of the PCL in the blends when contrasted with the non-compatibilized blends. The *X_c_* of PP decreased in all the compatibilized blends when contrasted with that of blends without compatibilizer. Similar to the PP/PCL blends without compatibilizer, PP80PCL20 had the highest *X_c_*, whereas PP90PCL10 and PP70PCL30 had somewhat lower *X_c_*, indicating that adding 20 wt.% PCL improves the crystallization of the PP segment slightly.

**Figure 3:**
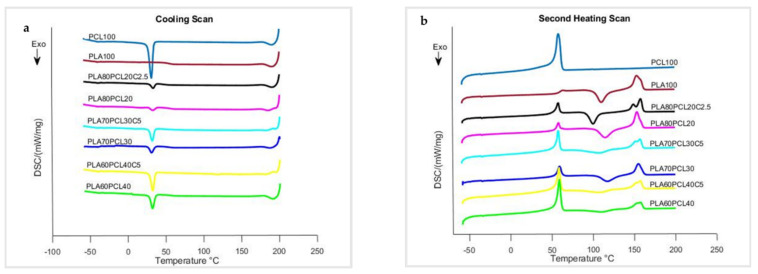
DSC curves of PLA, PCL, PLA/PCL, and blends with additive: (**a**) cooling scans and (**b**) second heating scans.

Results obtained from DSC for PLA100, PCL100, and selected blends are shown in Figure 3a,b as cooling and heating scans, while the thermal parameters obtained are summarized in Table 3. PCL100 displayed melting crystallization (T_mc_) peaks at 32.3 °C (Figure 3a), while PLA100 displayed a cold crystallization (T_cc_) peak at 115.2 °C (Figure 3b). PCL100 and PLA100 had melting temperatures (T_m_) at 58.6 °C and 153.6 °C, respectively (Figure 3a). T_mc, PCL_ did not change much with varying PCL content and stayed at approximately 33 °C. Both PLA100 and PLA in blends showed cold crystallization peaks rather than melt crystallization peaks due to the slow crystallization rate of PLA [17]. T_cc_, PLA increased slightly from 111.5 °C for PLA100 to a range of 115.7–118.6 °C for PLA/PCL with 20–40 wt.% PCL. T_m, PLA_ and T_m, PCL_ of PCL did not change much with varying PCL content, and their values stayed around 154 °C and 59 °C, respectively.

The lack of or only minor changes in the PLA and PCL thermal transitions in the blends indicates little or no interaction between the two polymers, implying poor miscibility [28]. Figure 3b shows the T_g_ of PLA from PLA100 and PLA90PCL10 plots. T_g_ was not visible for other blends with higher PCL concentration because of overlap with the T_m_ of PCL. The melt crystallization enthalpy (*H_mc_*) of PCL100 and cold crystallization enthalpy (*H_cc_*) of PLA100, given in Table 3, were 57.3 Jg^−1^ and 26.6 Jg^−1^, while their melting enthalpy (H_m_) was 65.8 Jg^−1^ and 25.9 Jg^−1^, respectively. The *H_mc_* and *H_m_* of PCL in PLA/PCL blends increased as the PCL content increased, but the *H_cc_* and *H_m_* of PLA in the blends decreased as the PCL content increased. The degree of crystallization (*X_c_*) of PLA100 and in blends was determined using Equation (2). *X_c_* of 48.38% and 0% for PCL100 and PLA100, respectively, shows that the PCL exhibited excellent crystallization behavior, but the PLA did not crystallize. *X_c_*_, PLA_ increased gradually, although not visibly, with increasing PCL content, indicating that PCL content enhances PLA crystallization. *X_c_*, _PCL_ of PLA80PCL20 was determined as 15%, while that of PLA60PCL40 was calculated to be 33.2% and nearly the *X_c_* of PCL100. These *X_c_*_, PCL_ results indicate that PCL has an excellent crystallization capability that is limited at low PCL concentrations. Figure 3 and Table 3 also show the results for blends with the P-123 additive. T_mc, PCL_ (Figure 3a) and T_m, PCL_ (Figure 3b) did not change considerably with changing PCL content and the inclusion of P-123 and remained at approximately 33 °C and 59 °C, respectively. T_cc, PLA_ (Figure 3a) and Tm, PLA (Figure 3b) both changed with changing PCL content and the addition of P-123, while T_m, PLA_ had double-melting behavior. The change in T_m_, _PLA_ could be because of good interaction between the two polymers, whilst the double-melting behavior could be because of the formation of distinct crystal structures, with the lower melting temperature corresponding to β-crystals and the higher melting temperature relating to α-crystals [31]. As shown in Table 3, the *H_mc_* and *H_m_* of PCL in PLA/PCL/P-123 blends increased as the PCL content increased, and it also increased compared to blends without additive. The *H_cc_* and *H_m_* of PLA in the blends decreased as the PCL content increased. The additive P-123 significantly improved the crystallization (*X_c_*) behavior of PCL and PLA in the blends when compared to the blends without additive. *X_c,_*_PLA_ increased as PCL content increased, demonstrating that PCL content enhances PLA crystallization. *X_c_*_, PCL_ of PLA80PCL20C2.5 was calculated to be 27.9%, while that of PLA60PCL40C5 was calculated to be 45.7% and about the same as *X_c_* of PCL100. These *X_c_*_, PCL_ results indicate that PCL has excellent crystallization ability that is limited at low PCL concentrations.

### 3.5. Morphology of Blends

**Figure 4:**
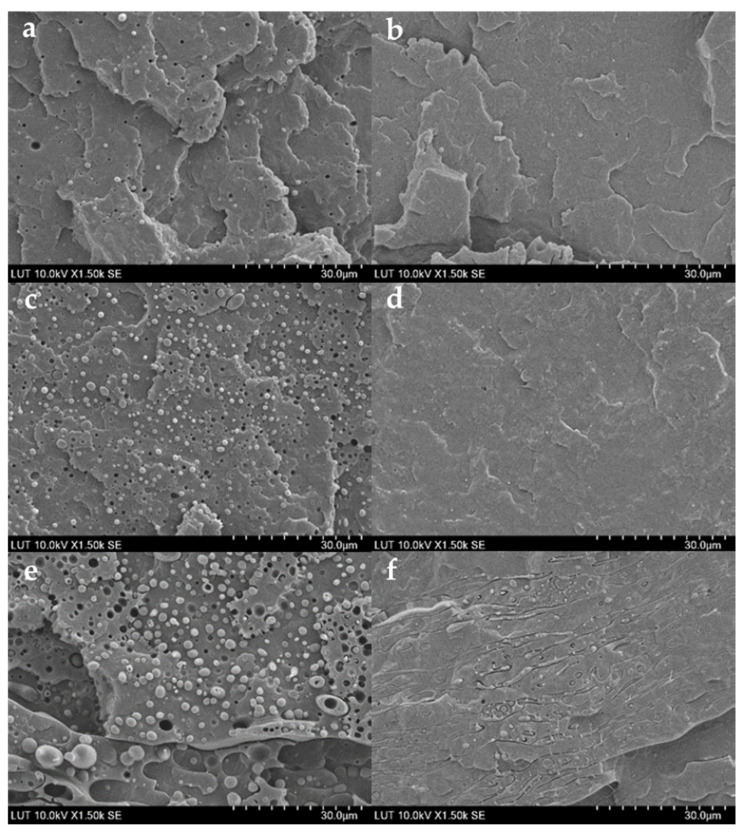
SEM images of (**a**) PP90/PCL10, (**b**) PP90/PCL10C1.25, (**c**) PP80/PCL20, (**d**) PP80/PCL20C1.25, (**e**) PP70/PCL30, and (**f**) PP70/PCL30C1.25 [30].

Figure 4 shows SEM micrographs of PP/PCL (10–30 wt.%) and PP/PCL compatibilized blends taken from their cryogenic fractured surfaces. As can be seen in Figure 4a,c,e, PP/PCL blends have a phase-separated morphology and are not miscible. The micrograph shows spherical PCL droplets and holes scattered throughout the PP matrix. The size of the spherical droplets and holes increased as the PCL content in the blends increased. An almost smooth structure with few small holes and spherical droplets of PCL was observed when 1.25 phr of PP-g-ma was added to PP/PCL blends. The almost smooth structures obtained when PP90/PCL10 and PP80/PCL20 blends were compatibilized are shown in Figure 4b,d. PP80/PCL20C1.25 had slightly more spherical droplets of PCL and holes than PP90/PCL10C1.25 because of its higher PCL content. Figure 4f shows that PP70/PCL30C1.25 with the highest PCL content had larger spherical droplets of PCL than PP80/PCL20C1.25 and even showed fibrous forms of PCL. This finding indicates that the compatibilizer enhanced the miscibility and interfacial adhesion of both phases. While raising the concentration of the compatibilizer will enhance the miscibility and interfacial adhesion in blends even more, tests revealed that the ductility will suffer significantly (Table S2).

**Figure 5:**
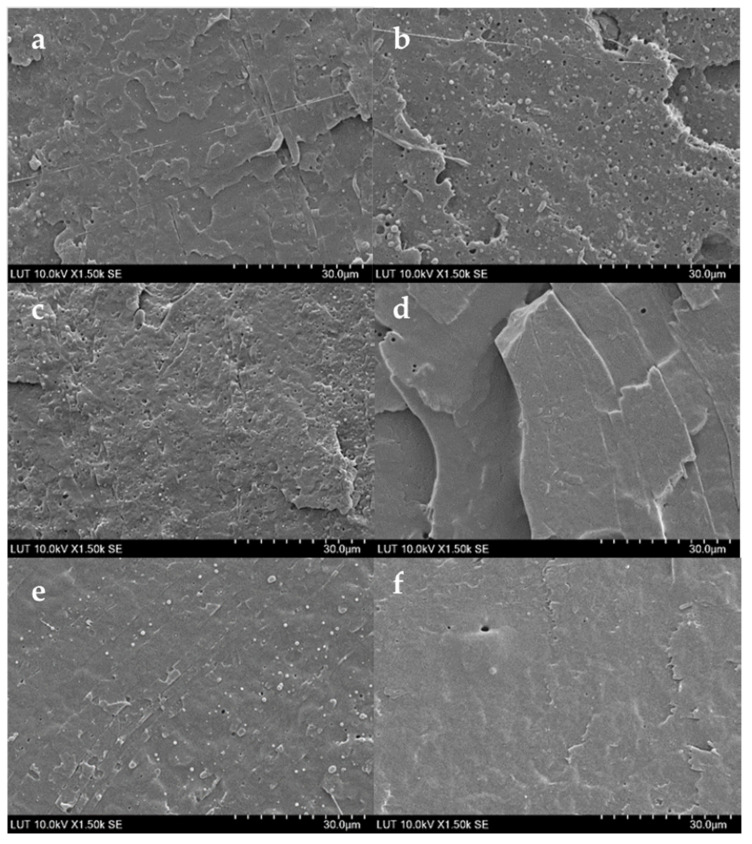
SEM images of (**a**) PLA80/PCL20, (**b**) PLA80/PCL20C2.5, (**c**) PLA70/PCL30, (**d**) PLA70/PCL30C5, (**e**) PLA60/PCL40, and (**f**) PLA 60/PCL40C5 [30].

Figure 5 shows SEM micrographs of PLA/PCL (20–40 wt.%) and PLA/PCL compatibilized blends taken from their cryogenic fractured surfaces. As can be seen in Figure 5a,c,e, PLA/PCL blends have a phase-separated morphology and are not miscible. The micrograph shows spherical PCL droplets and holes scattered throughout the sea–island structure of the PLA matrix. The number of spherical droplets increased as the PCL content in the blends increased from 20 wt.% to 30 wt.%, and PCL droplets became elongated in the PLA60/PCL40 blend. This blend displayed a semi-continuous PCL phase and a small number of PCL spheres. An almost smooth structure with a few small holes and spherical droplets of PCL was observed when 5 phr of P-123 was added to PLA70/PCL30 and PLA60/PCL40 blends as shown in Figure 5d,f, respectively, indicating that the additive enhanced the miscibility and interfacial adhesion of both phases. This contrasts with the findings of Wachirahuttapong et al. [29] where the additive merely served as a plasticizer and did not increase miscibility. The micrograph of the PLA80/PCL20 blend with 2.5 phr additive shown in Figure 5b revealed larger PCL droplets and more spherical PCL droplets, which could be attributed to the additive’s low concentration. To verify this, 5 phr of P-123 was added to the PLA80/PCL20 blend, and it showed fewer spherical droplets, as shown in Supporting Information (Figure S3).

### 3.6. Shape Memory Behavior

**Figure 6:**
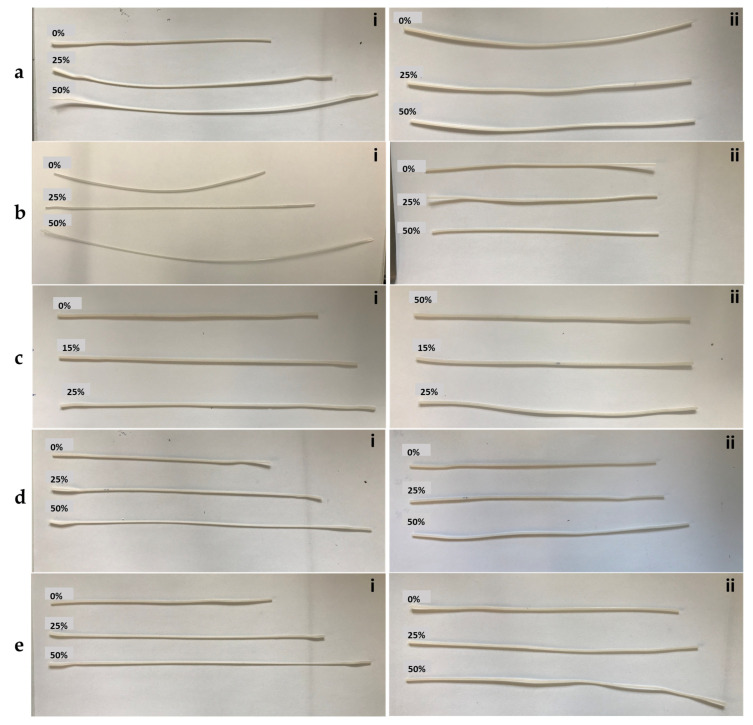
Thermomechanical cycles: (**a**) PLA70PCL30 blend at 0, 25, and 50%: (i) fixing of the temporary shape; (ii) recovery of the permanent shape. (**b**) PLA60PCL40 blend at 0, 25, and 50%: (i) fixing of the temporary shape; (ii) recovery of the permanent shape. (**c**) PLA80PCL20C2.5 blend at 0, 15, and 25%: (i) fixing of the temporary shape; (ii) recovery of the permanent shape. (**d**) PLA70PCL30C5 blend at 0, 25, and 50%: (i) fixing of the temporary shape; (ii) recovery of the permanent shape. (**e**) PLA60/PCL40C5 blend at 0, 25, and 50%: (i) fixing of the temporary shape; (ii) recovery of the permanent shape [30].

Thermo-responsive shape memory behavior of the PP/PCL and PLA/PCL blends was examined at different strains. These blends were chosen for shape memory analysis based on the findings of the morphology, DSC, tensile, and 3D-printing tests. Table 4 shows calculated *R_r_* and *R_f_* values for the PLA/PCL blends, and Figure 6 shows a series of images taken to demonstrate the shape memory process. The test specimens were heated, deformed, and then cooled to fix the temporary shape. After being heated again, the deformed samples regained their original shape. All the investigated PLA/PCL blends and those with additive displayed substantial shape memory behavior throughout the cycle, as can be seen in Table 4 and Figure 6. *R_f_* values were high in all the blends (>98%). The *R_r_* values of the blends without additive were higher (>97%) than those of the blends with additive (61–96%). The *R_r_* values were observed to decrease as the content of PCL in the blends increased from 30 to 40 wt.%. The *R_r_* value was about 99% for the PLA70/PCL30 blend and the highest in all the blends. After testing the samples over three cycles, no substantial change was observed.

The blends were stretched at various strains to study the impact of temporary strain on *R_r_* and *R_f_* values. Strains were chosen based on the elongation characteristics of the blends. Results are given in Table 4, where it can be seen that the *R_r_* and *R_f_* values of PLA/PCL blends without additive were unaffected by the temporary strain, whereas the R_r_ values of blends with additive decreased. *R_r_* time for PLA/PCL blends without additive was 10–15 min, while it was 10–20 min for PLA/PCL blends with additive.

To understand the shape memory process better, the morphology, melting, and crystallization of the blends were studied. The DSC results revealed that the PLA phase acted as the net points (hard segment) during the shape memory cycle while the PCL phase with the lower T_m_ acted as the switching segment (soft segment). After the samples had been heated at a temperature lower than T_m, PLA_ but a little higher than T_m, PCL_, the crystals of PCL in the blends melted and their molecular chains transitioned to a temporary shape upon the application of strain. The molecular chain of PLA with a higher T_m_ remained frozen at this temperature and functioned as a fixed phase responsible for keeping the permanent shape. The high *R_f_* values of the blends can be attributed to the excellent crystallization behavior of PCL. Cooling of the samples was accompanied by rapid crystallization of the molecular chains of the PCL and subsequent fixing of the temporary shape with no external force required. DSC studies showed that *X_c_*_, PCL_ increased as PCL concentration in the blends increased, suggesting that additional PCL crystals participated in fixing the temporary shape. As shown in Table 4, *R_r_* values reduced as the PCL concentration increased. This could be because of physical crosslinking at interfaces, which is crucial for shape recovery. Liu et al. [17] came to the same conclusion for PLA/PCL blends. As PCL concentration increases in blends, physical crosslinking at the interfaces that control the viscous flow of PCL molecular chains reduces, leading to irreversible molecular chain slippage and, as a result, a reduction in the entropy elasticity recovery of the PCL [17]. SEM results confirmed that the evenly dispersed PCL phase was totally encircled by the PLA phase in the PLA70/PCL30 blend, forcing the molecular chains to flow in a constrained space, whereas the interfaces between the semi-continuous PLA and PCL phases were unstable in the PLA60/PCL40 blend. Physical crosslinking at the interfaces could control the viscous flow of PCL molecular chains, causing slight irreversible molecular chain slippage for the PLA70/PCL30 blend and greater chain slippage for the PLA60/PCL40 blend.

Thermo-responsive shape memory behavior was not seen in all the PP/PCL blends and blends with compatibilizer. This could be because the crystals of PCL in the PP/PCL blends did not melt and their molecular chains did not transition to a temporary shape upon the application of strain, after the samples had been heated at a temperature lower than T_m, PP_ but slightly higher than T_m, PCL_.

### 3.7. The 3D-Printed Respirator Mask

**Figure 7:**
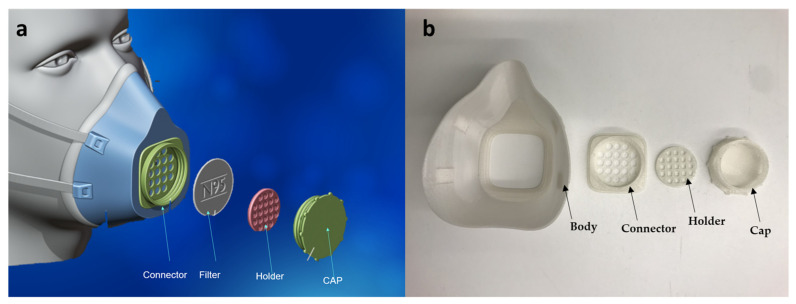
The respirator mask: (**a**) 3D model; (**b**) printed mask displaying the permanent shape (before molding).

The printed mask is made up of four parts: the cap, filter connector, filter holder, and mask body. PLA100 filament was used to print the cap, the filter holder, and the filter connector, while shape memory PLA70PCL30 filament was used to print the mask body, which is responsible for the mask fit. PLA70PCL30 was chosen for its mechanical qualities as well as its excellent R_r_ and R_f_ values. Figure 7a shows an image of the 3D model of the respirator mask from MakerBot Thingiverse [26]. Figure 7b shows an image of the printed mask before fitting, and Table S7 (Supporting Information) gives the printing parameters used to print the mask.

### 3.8. Respirator Mask Fitting

**Figure 8:**
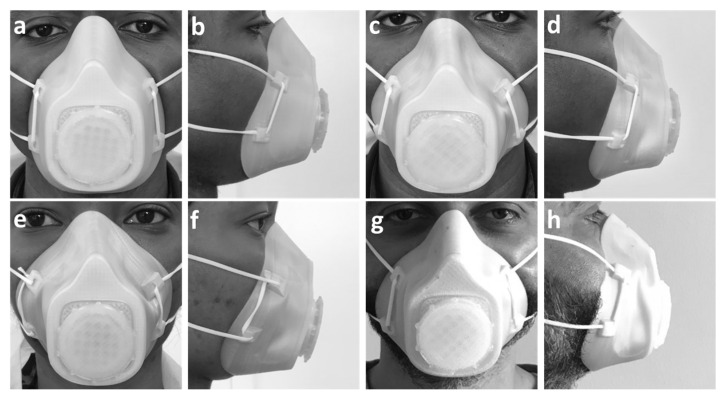
Mask fitting: (**a**,**b**) Front and side view of original mask on first fitter; (**c**,**d**) front and side view of molded mask on first fitter; (**e**,**f**) front and side view of molded mask on second fitter; (**g**,**h**) front and side view of molded mask on third fitter [30].

The ability of the 3D-printed respirator mask to change shape, mold, and remold to fit various face shapes and sizes was explored. The mask was heated in an oven for 10 min at 65 °C to thermally activate it. Then it was swiftly placed on the fitter’s face for about 3–5 min to take shape before being removed. The same mask was thermally activated and molded to various fitters. Figure 8 shows photos of the mask before and after fitting on several people. Table 5 shows the mask’s dimensions before and after molding to various fitters.

### 3.9. Self-Healing

**Figure 9:**
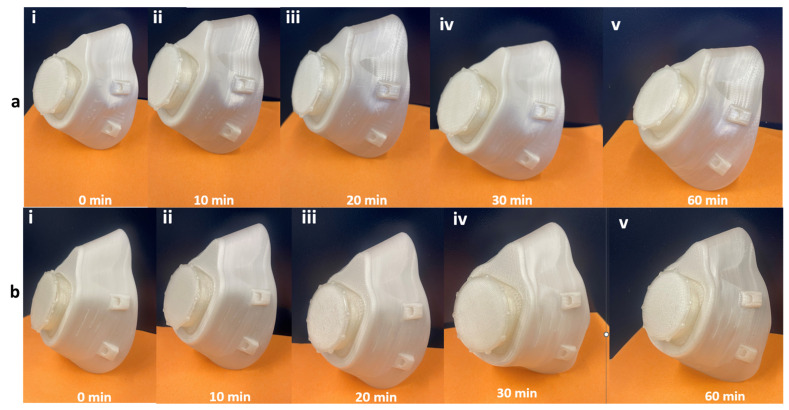
Self-healing process of the 3D-printed respirator mask: (**a**) from scratches ((i) 0 min, (ii) 10 min, (iii) 20 min, (iv) 30 min, (v) 60 min); (**b**) from slashes ((i) 0 min, (ii) 10 min, (iii) 20 min, (iv) 30 min, (v) 60 min).

The 3D-printed respirator mask was examined for self-healing. A razor was used to place 1 cm, 1.5 cm, and 2 cm long slashes and scratches on the mask. The deformed mask was activated at different times ranging from 0 to 60 min at 65 °C and then left to cool to room temperature. Figure 9 shows a series of images taken to demonstrate the self-healing process. It can be seen in Figure 9a that the scratches on the mask healed slightly. This self-healing is attributed to the reversible plasticity SME of SMPs and is aided by the flow and redistribution of molten PCL into the scratched surface. The slashes on the mask did not heal completely, but the sides of the opening from the slash came in close contact with each other, reducing the length and width of each slash by 0.5 cm. This could be because the slashed surfaces were close enough to each other for molten PCL to flow into the gap. Xie [22] reported that heating will mitigate irreversible damage such as crack width but will not repair the crack. Bhattacharya et al. [21] on the other hand reported achieving self-healing on fractured surfaces of TPU/PCL blends.

## 4. Conclusions

Shape memory polymers (SMPs) were evaluated as an alternative material for creating respirator masks that are able to alter their shape, mold, and remold to fit various face shapes and sizes. In this study, blends of two polymers, such as PLA/PCL, PP/PCL, and LDPE/PCL, with and without compatibilizers or additives were prepared into filaments by extrusion.

The mechanical properties of the blends were characterized, and it was found that PLA/PCL blends had the largest tensile strength and elastic modulus but had the lowest elongation at break, whereas LDPE/PCL had the lowest tensile strength and elastic modulus but had high elongation at break. The compatibilizer increased the tensile strength and elastic modulus of LDPE/PCL and PP/PCL blends while significantly reducing the ductility of the PP/PCL blend. The additive had little effect on the tensile strength and elastic modulus of PLA/PCL blends; however, it considerably increased the ductility. DSC revealed that PCL demonstrated good crystallization behavior which increased with increasing PCL content in the PLA and PP blends. All the polymer blends had low miscibility, which improved with the inclusion of a compatibilizer and additive. SEM showed phase separation in the morphology of all the blends, supporting the immiscibility predicted by DSC. Enhanced miscibility was detected following the addition of the compatibilizer or additive, showing the ability of the compatibilizer and additive to improve the interaction between the polymer interfaces. Three-dimensional printing tests were performed to confirm the suitability of the produced filaments. The PLA/PCL and PP/PCL filaments performed well in this study, but the LDPE/PCL filaments had low tensile strength and caused printing difficulties. Therefore, SM experiments were performed on PP/PCL and PLA/PCL blends at various strains, where PCL functioned as the reversible phase and the soft segment in the PLA/PCL blend, and PLA functioned as the fixity phase and hard segment. The deformation temperature was selected to be a little higher than the melting temperature of the PCL.

The PLA70/PCL30 blend was found to be the optimal SMP and 3D-printing polymer for the respirator mask based on all characterization tests. After printing, the masks made from this blend were activated by heating and molded to various faces to examine shape fixing and recovery. The masks demonstrated good SM over several molding and remolding cycles. In addition, the masks’ ability to self-heal was studied. Scratches and slashes on the surface of the mask healed slightly. The sides of the opening from the slash came in close contact with each other, reducing the width of the slash.

Overall, this study demonstrates the potential of 3D-printable SMPs to create respirator masks that can adapt to different face shapes and sizes. This approach could have practical applications in the production of respirator masks that can fit any face shape and size, providing a more effective and comfortable means of protection.

## Figures and Tables

**Table 1:** Materials used and their properties.

Material	M_w_/M_n_	Density (g/cm^3^)	MFI (g/10 min)	T_m_ (°C)	Form	Purchased
Polylactic acid (PLA)	-	1.24	6	175	Filament	3D-CAD solution
Polycaprolactone (PCL)	M_n_~80,000	1.15	2.01–4.03 (160 °C/5 kg)	60	Pellets (~3 mm)	Sigma-Aldrich
Polypropylene (PP)	M_w_~340,000 Mn~97,000	0.90	4 (230 °C/2.16 kg)	160–165	Pellets	Sigma-Aldrich
Low-density polyethylene (LDPE)		0.92	1 (190 °C/2.16 kg)	100–125	Pellets	Sigma-Aldrich
Polyethylene-graft-maleic anhydride (PE-g-ma)		0.92		107	Pellets	Sigma-Aldrich
Polypropylene-graft-maleic anhydride (PP-g-ma)	Mw~9100 Mn~3900	0.93		156	Pellets	Sigma-Aldrich
Pluronic P-123 (P-123)	Mn~5800	-		39	Semi-solid	Sigma-Aldrich

**Table 2:** Thermal parameters of PP, PCL, PP/PCL, and PP/PCL/PP-g-ma [30].

Sample	PP					PCL				
	T_mc_ (°C)	T_m_ (°C)	*H_mc_* (Jg^−1^)	*H_m_* (Jg^−1^)	*X_c_* (%)	T_mc_ (°C)	T_m_ (°C)	*H_mc_* (Jg^−1^)	*H_m_* (Jg^−1^)	*X_c_* (%)
PP100	115.20	163.80	82.59	78.33	38.21	-	-	-	-	-
PP90PCL10	116.30	163.70	76.53	72.02	39.04	35.35	57.57	2.93	2.09	15.35
PP80PCL20	118.20	163.80	71.93	68.69	41.88	35.31	57.67	7.11	6.40	23.51
PP70PCL30	115.30	162.70	61.88	57.37	39.98	34.34	58.59	11.42	11.27	27.62
PP90PCL10C1.25	115.40	163.60	75.29	70.95	38.46	31.45	57.51	3.18	2.79	20.50
PP80PCL20C1.25	114.20	165.80	71.65	66.08	40.29	32.31	58.67	6.37	5.74	21.11
PP70PCL30C1.25	113.30	165.70	59.95	55.52	38.69	30.34	59.63	12.65	11.39	27.92
PCL100	-	-	-	-	-	32.33	58.64	57.31	65.80	48.38

**Table 3:** Thermal parameters of PLA, PCL, PLA/PCL, and PLA/PCL/ PEG-PPG-PEG [30].

Sample	PLA						PCL				
	T_g_ (°C)	T_m_ (°C)	T_mc_ (°C)	*H_m_* (Jg^−1^)	*H_mc_* (Jg^−1^)	*X_c_* (%)	T_m_ (°C)	T_mc_ (°C)	*H_m_* (Jg^−1^)	*H_mc_* (Jg^−1^)	*X_c_* (%)
PLA100	60	153.6	111.5	25.9	26.6	-0.7	-	-	-	-	-
PLA80PCL20	-	153.8	115.7	22.9	23.2	-0.5	58.7	33.3	4.1	4.7	15.1
PLA70PCL30	-	154.7	117.7	16.6	16.9	-0.4	59.6	31.3	8.7	9.7	21.3
PLA60PCL40	-	154.7	118.6	14.3	13.9	0.8	58.6	32.3	18.1	20.7	33.2
PLA80PCL20C2.5	-	149.7/158	100.6	21.9	20.0	2.4	58.6	34.3	7.6	6.0	27.9
PLA70PCL30C5	-	151.7/157	107.6	15.8	10.2	8.7	57.6	32.4	16.0	16.4	39.3
PLA60PCL40C5	-	150.7/157	102.7	11.5	4.3	12.7	58.7	32.4	24.8	24.7	45.7
PCL100	-	-	-	-	-	-	58.6	32.3	65.8	57.3	48.4

**Table 4:** Shape fixity and shape recovery values of studied PLA/PCL blends [30].

Sample	Strain at 15%		Strain at 25%		Strain at 50%	
	*R_f_* (%)	*R_r_* (%)	*R_f_* (%)	*R_r_* (%)	*R_f_* (%)	*R_r_* (%)
PLA70PCL30	-	-	100	99.99	100	99.45
PLA60PCL40	-	-	99.56	99.99	100	98.88
PLA80PCL20C2.5	100	96.40	100	89.12	-	-
PLA70PCL30C5	-	-	100	80	100	72.22
PLA60PCL40C5	-	-	100	73.76	99.63	61.74

**Table 5:** Mask dimensions before and after molding to various fitters [30].

Dimensions	3D-Printed Mask	First Fitter	Second Fitter	Third Fitter
Length (mm)	89.8	80.6	81.2	82.9
Depth (mm)	58.5	50.4	53.1	54.2
Width (mm)	70.7	76.1	76.1	79.6

## Data Availability

Not applicable.

## Supplementary Materials

The following supporting information can be downloaded at: https://www.mdpi.com/article/10.3390/polym15081859/s1, Table S1: Summary of the mechanical properties of PP/PCL blends obtained from tensile tests. Table S2: Summary of the mechanical properties of PP/PCL/PP-g-ma blends obtained from tensile tests. Table S3: Summary of the mechanical properties of PLA/PCL blends obtained from tensile tests. Table S4: Summary of the mechanical properties of PP/PCL/P-123 blends obtained from tensile tests. Table S5: Summary of the mechanical properties of LDPE/PCL blends and LDPE/PCL/PE-g-ma blends obtained from tensile tests. Figure S1: DSC plots of LDPE, PCL, and LDPE/PCL compatibilized blends: (a) cooling scans and (b) second heating scans. Table S6: Thermal parameters of LDPE, PCL, LDPE/PCL, and LDPE/PCL/ PE-g-ma. Figure S2: SEM images of (a) LDPE90/PCL10, (b) LDPE90/PCL10C10, (c) LDPE80/PCL20, (d) LDPE80/PCL20C10, (e) LDPE70/PCL30, and (f) LDPE70/PCL30C10. Figure S3: SEM image of PLA80/PCL20C5. Table S7: Printing parameters used in printing mask.
