# Supplementary material for: Shape Memory Respirator Mask for Airborne Viruses
Source: Polymers (Basel). 2023 Apr 13;15(8):1859. doi: 10.3390/polym15081859 (PMC10145067; doi:10.3390/polym15081859)
Supplement: Supplementary file 1 [file polymers-15-01859-s001.zip › polymers-2248591-supplementary.pdf]

Supporting Information

# Shape Memory Respirator Mask for Airborne Viruses

Kosisochi Ibebunjo [1,*], Susanna Tella [2], Samantha Kiljunen [1] and Eveliina Repo [1]

[1] School of Engineering Science, Department of Separation Science, LUT University, FI-53850 Lappeenranta, Finland
[2] Faculty of Health Care and Social Services, LAB University of Applied Sciences, FI-53850 Lappeenranta, Finland
* Correspondence: kosisochi.ibebunjo@lut.fi

**Table S1.** Summary of the mechanical properties of PP/PCL blends obtained from tensile tests [30].

| Sample | Elastic Modulus (MPa) | Tensile Strength (MPa) | Elongation at Break (%) |
|---|---|---|---|
| PP100 | 1866 ± 350 | 38.3 ± 2.3 | 926 ± 1 |
| PP90PCL10 | 1234 ± 70 | 28.5 ± 4.2 | 926 ± 1 |
| PP80PCL20 | 1117 ± 80 | 25.4 ± 1.3 | 926 ± 1 |
| PP70PCL30 | 890 ± 80 | 27.6 ± 6 | 549 ± 247 |
| PCL100 | 327.4 ± 10 | 19.3 ± 1.5 | 926 ± 1 |

**Table S2.** Summary of the mechanical properties of PP/PCL/PP-g-ma blends obtained from tensile tests [30].

| **Sample** | **Elastic Modulus (MPa)** | **Tensile Strength (MPa)** | **Elongation at Break (%)** |
|---|---|---|---|
| PP90PCL10C10 | 1233 ± 90 | 30.2 ± 1.5 | 32.7 ± 26.3 |
| PP90PCL10C5 | 1540 ± 268 | 30.7 ± 5.38 | 98.9 ± 51.7 |
| PP90PCL10C1.25 | 1356 ± 77 | 32.3 ± 1.1 | 104.3 ± 32.4 |
| PP80PCL20C10 | 1160 ± 140 | 30.1 ± 0.6 | 65.8 ± 9.7 |
| PP80PCL20C5 | 1457 ± 106 | 30.7 ± 5.7 | 178.8 ± 158 |
| PP80PCL20C1.25 | 1402 ± 46 | 33.5 ± 0.8 | 80.3 ± 12.5 |
| PP70PCL30C10 | 1152 ± 140 | 27.7 ± 3.8 | 134 ± 46.4 |
| PP70PCL30C5 | 1424 ± 173 | 31.4 ± 3.6 | 69.3 ± 15.7 |
| PP70PCL30C1.25 | 1307 ± 52 | 32.0 ± 1.4 | 116.8 ± 36.3 |

**Table S3.** Summary of the mechanical properties of PLA/PCL blends obtained from tensile tests [30].

| **Sample** | **Tensile Strength (MPa)** | **Elastic Modulus (MPa)** | **Elongation at Break (%)** |
|---|---|---|---|
| PLA100 | 58.4 ± 4.3 | 2883 ± 220 | 8.62 ± 3.9 |
| PLA90PCL10 | 52.8 ± 9.3 | 3087 ± 440 | 13.2 ± 2.0 |
| PLA80PCL20 | 44.5 ± 1 | 2628 ± 76 | 17.9 ± 7.5 |
| PLA70PCL30 | 37.8 ± 4.7 | 2252 ± 73 | 535 ± 0.3 |
| PLA60PCL40 | 32.8 ± 5.7 | 1989 ± 510 | 127 ± 99.2 |
| PLA50PCL50 | 25.2 ± 3.5 | 1604 ± 276 | 225.2 ± 78.7 |
| PCL100 | 19.3 ± 1.5 | 327.4 ± 10 | 926 ± 1 |

**Table S4.** Summary of the mechanical properties of PP/PCL/P-123blends obtained from tensile tests [30].

| Sample | Tensile Strength (MPa) | Elastic Modulus (MPa) | Elongation at Break (%) |
|---|---|---|---|
| PLA80PCL20C2.5 | 39.3 ± 20.3 | 2555 ± 216 | 54.7 ± 38.9 |
| PLA80PCL20C5 | 45.3 ± 2.6 | 2710 ± 170 | 41.0 ± 34.4 |
| PLA80PCL20C7.5 | 41.7 ± 1.9 | 2609 ± 285 | 25.6 ± 9.1 |
| PLA80PCL20C10 | 33.5 ± 9.4 | 2172 ± 464 | 45.9 ± 65.4 |
| PLA70PCL30C5 | 33.96 ± 2.3 | 1814 ± 184 | 108.1 ± 38.0 |
| PLA70PCL30C7.5 | 32.2 ± 3.28 | 2001 ± 136 | 43.9 ± 35.4 |
| PLA70PCL30C10 | 31.3 ± 5.9 | 2110 ± 270 | 50.6 ± 35.9 |
| PLA60PCL40C5 | 31.3 ± 3.9 | 1691 ± 276 | 535.6 ± 355.1 |
| PLA60PCL40C7.5 | 37.6 ± 2.7 | 1958 ± 229 | 137.2 ± 67.1 |
| PLA60PCL40C10 | 34.8 ± 5.6 | 1769 ± 113 | 119.9 ± 56.4 |

**Table S5.** Summary of the mechanical properties of LDPE/PCL blends and LDPE/PCL/PE-g-ma blends obtained from tensile tests [30].

| Sample | Tensile Strength (MPa) | Elastic Modulus (MPa) | Elongation at Break (%) |
|---|---|---|---|
| LDPE100 | 12.9 ± 1.7 | 289.5 ± 50 | 926 ± 1 |
| LDPE90PCL10 | 11.6 ± 2.1 | 250.1 ± 20 | 926 ± 1 |
| LDPE80PCL20 | 12.7 ± 1.1 | 284.3 ± 40 | 926 ± 1 |
| LDPE70PCL30 | 12.8 ± 1.4 | 340.6 ± 70 | 926 ± 1 |
| LDPE90PCL10C10 | 11.2 ± 15 | 274.9 ± 30 | 926 ± 1 |
| LDPE80PCL20C10 | 12.1 ± 1.1 | 295.7 ± 20 | 926 ± 1 |
| LDPE70PCL30C10 | 12.4 ± 1.1 | 353.7 ± 50 | 488 ± 302 |
| PCL100 | 19.3 ± 1.5 | 327.4 ± 10 | 926 ± 1 |

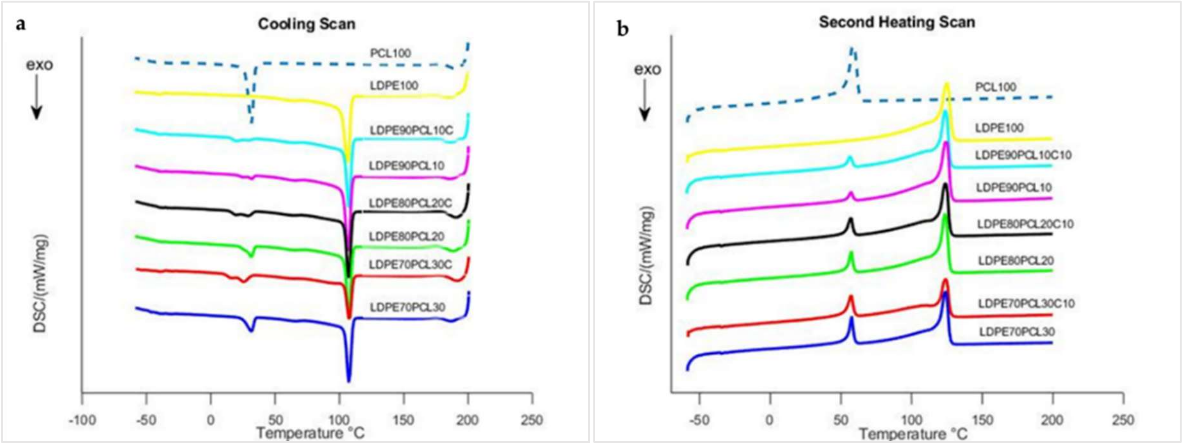


**Figure S1.** DSC plots of LDPE, PCL, LDPE/PCL compatibilized blends: (**a**) Cooling scans and (**b**) Second heating scans.

**Table S6.** Thermal parameters of LDPE, PCL, LDPE/PCL, and LDPE/PCL/ PE-g-ma [30].

| **Sample** | **LDPE** | | | | | **PCL** | | | | |
|---|---|---|---|---|---|---|---|---|---|---|
| | **$T_m$ (◦C)** | **$T_{mc}$ (◦C)** | **$H_m$ (Jg$^{-1}$)** | **$H_{mc}$ (Jg$^{-1}$)** | **$X_c$ (%)** | **$T_m$ (◦C)** | **$T_{mc}$ (◦C)** | **$H_m$ (Jg-1)** | **$H_{mc}$ (Jg-1)** | **$X_c$ (%)** |
| LDPE100 | 125.7 | 107.2 | 99.85 | 103.70 | 34.08 | - | - | - | - | - |
| LDPE90PCL10 | 124.6 | 107.3 | 93.41 | 95.23 | 35.42 | 57.55 | 32.38 | 3.04 | 2.86 | 22.37 |
| LDPE80PCL20 | 123.8 | 107.4 | 78.43 | 85.59 | 33.46 | 57.40 | 31.31 | 8.56 | 9.73 | 31.47 |
| LDPE70PCL30 | 123.7 | 107.2 | 71.18 | 77.21 | 34.71 | 57.67 | 31.32 | 11.06 | 13.08 | 27.11 |
| LDPE90PCL10C10 | 124.3 | 107.3 | 83.80 | 92.06 | 31.78 | 56.56 | 30.50 | 4.44 | 3.51 | 32.66 |
| LDPE80PCL20C10 | 124.2 | 107.3 | 75.62 | 87.13 | 32.26 | 57.59 | 29.32 | 8.01 | 8.35 | 29.46 |
| LDPE70PCL30C10 | 124.7 | 107.3 | 58.47 | 69.04 | 28.51 | 57.63 | 26.33 | 10.98 | 10.45 | 26.91 |
| PCL100 | - | - | - | - | - | 58.64 | 32.33 | 65.80 | 57.31 | 48.38 |

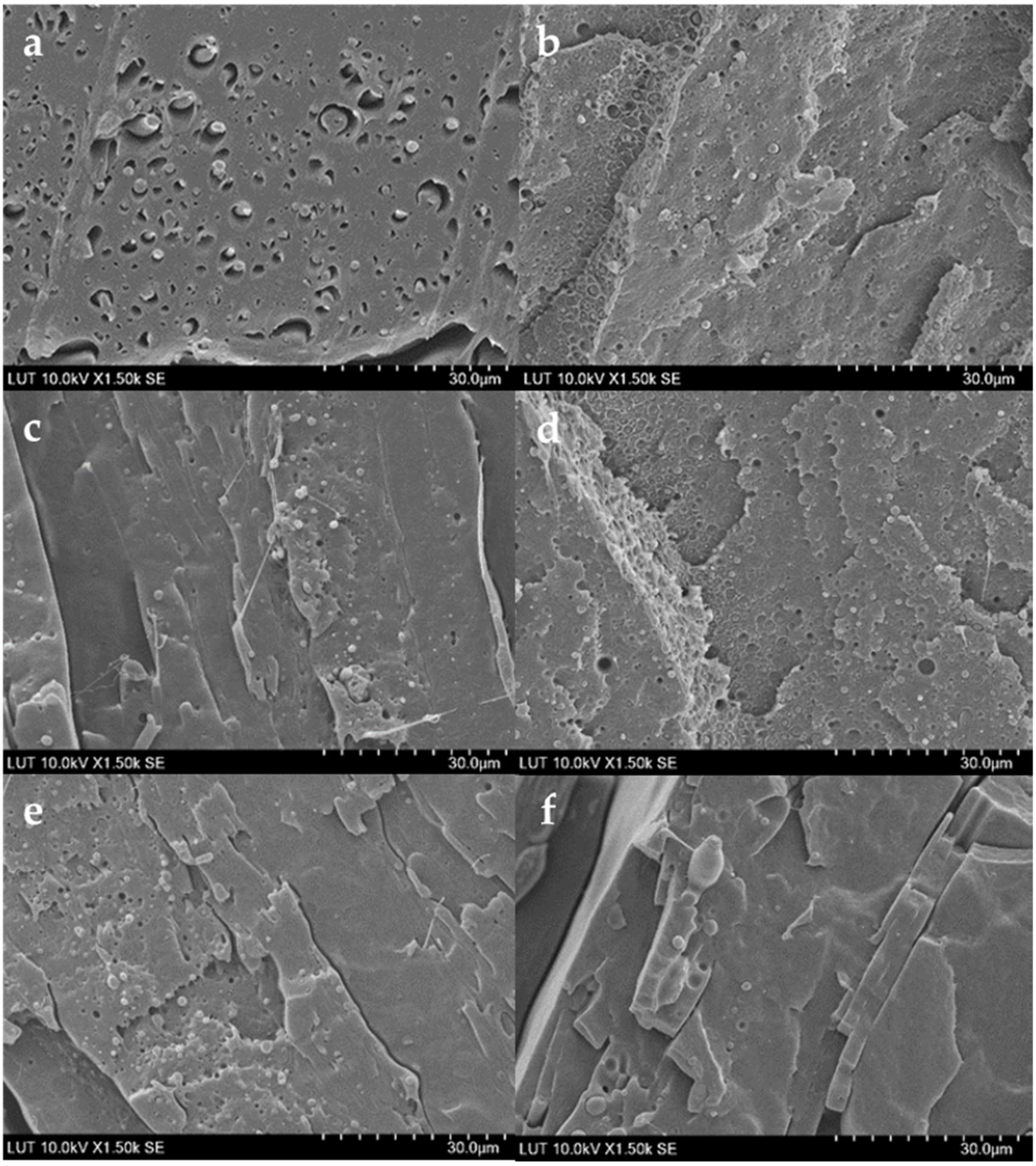


**Figure S2.** SEM images of: (**a**) LDPE90/PCL10 (**b**) LDPE90/PCL10C10 (**c**) LDPE80/PCL20 (**d**) LDPE80/PCL20C10 (**e**) LDPE70/PCL30 and (**f**) LDPE70/PCL30C10 [30].

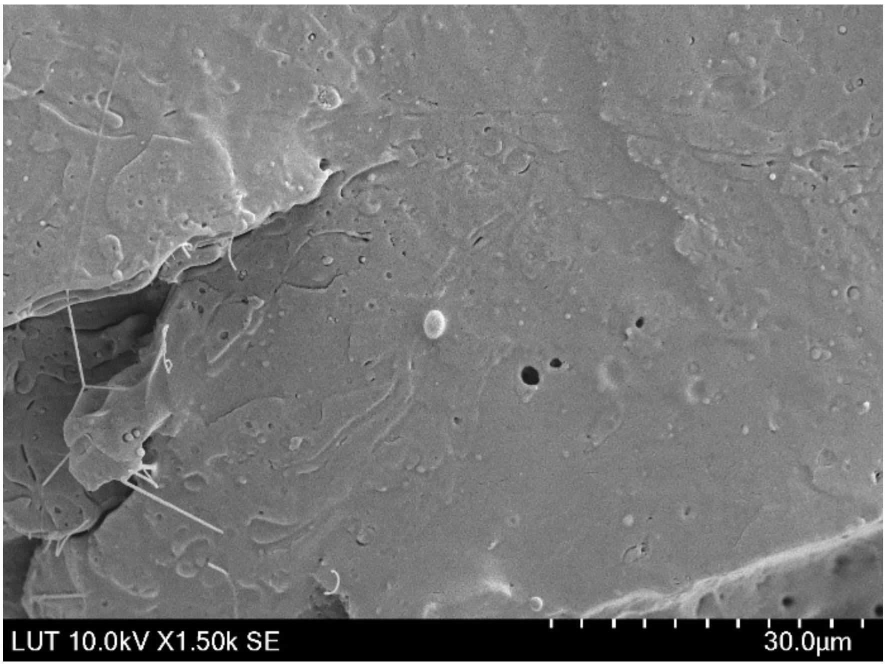


**Figure S3.** SEM image of PLA80/PCL20C5 [30].

**Table S7.** Printing parameters used in printing mask.

| Sample | Bed Temperature (°C) | Nozzle Temperature (°C) | Raster Angle (°) | Printing Speed (mm/min) | Infill Density (%) |
|---|---|---|---|---|---|
| Mask body | 50 | 205 | 45 | 6000 | 15 |
| Other parts | 50 | 210 | 45 | 6000 | 15 |
